# Hospital Meetings

**Published:** 1905-07-29

**Authors:** 


### HOSPITAL MEETINGS.

### THE EAST LONDON HOSPITAL FOR CHILDREN.

THE half-yearly Court of the Governors of this hospital was held in the Board Room on the 17th instant. Colonel Charles Needham took the chair, and in commenting on the financial difficulties under which the hospital was labouring, as shown in the report before the meeting, he made an earnest appeal to the Governors to use their best efforts to remove the distress. If this was not done, either (1) one of the wards, (2) the Convalescent Home at Bognor, or (3) the Women's Dispensary must be closed. As the hospital was intended solely for children, the last-named would probably be their best choice, but he hoped there would be no necessity to inflict such a great loss upon the poor at all. A long spell of infection in the wards has been responsible for a decreased number of in-patients, the figures of those admitted from January to June of this year being—In-patients, 645; out-patients, 15,892. £10,000 was altogether necessary to pay for the new Isolation Block, and strenuous efforts were made to obtain by June 30th the amount necessary to secure the £300 and £500 promised respectively by the Zunz trustees and the Drapers' Company. They were successful owing to the generous action of their treasurer, Mr. Prescott, who himself gave £500 of the £750 required. Only £150 now remained to be collected.

The adoption of the report was moved by Mr. Machin, seconded by Mr. Luttman Johnson, and unanimously carried. The election of Colonel Francis Charrington, C.M.G., as an honorary life governor was moved by the Chairman, seconded by Mr. Luttman Johnson, and unanimously adopted.

The proceedings then terminated.

